# Progress and gaps in reproductive health services in three humanitarian settings: mixed-methods case studies

**DOI:** 10.1186/1752-1505-9-S1-S3

**Published:** 2015-02-02

**Authors:** Sara E Casey, Sarah K Chynoweth, Nadine Cornier, Meghan C Gallagher, Erin E Wheeler

**Affiliations:** 1 Heilbrunn Department of Population and Family Health, Mailman School of Public Health, Columbia University, 60 Haven Ave, New York, NY 10032 USA; 2 University of New South Wales, High St, Kensington NSW 2052, Australia; 3 United Nations High Commissioner for Refugees, Rue de Montbrillant 94, 1201 Geneva, Switzerland

**Keywords:** Reproductive health, humanitarian, refugees, Burkina Faso, Democratic Republic of the Congo, South Sudan

## Abstract

**Background:**

Reproductive health (RH) care is an essential component of humanitarian response. Women and girls living in humanitarian settings often face high maternal mortality and are vulnerable to unwanted pregnancy, unsafe abortion, and sexual violence. This study explored the availability and quality of, and access barriers to RH services in three humanitarian settings in Burkina Faso, Democratic Republic of the Congo (DRC), and South Sudan.

**Methods:**

Data collection was conducted between July and October 2013. In total, 63 purposively selected health facilities were assessed: 28 in Burkina Faso, 25 in DRC, and nine in South Sudan, and 42 providers completed a questionnaire to assess RH knowledge and attitudes. Thirty-four focus group discussions were conducted with 29 members of the host communities and 273 displaced married and unmarried women and men to understand access barriers.

**Results:**

All facilities reported providing some RH services in the prior three months. Five health facilities in Burkina Faso, six in DRC, and none in South Sudan met the criteria as a family planning service delivery point. Two health facilities in Burkina Faso, one in DRC, and two in South Sudan met the criteria as an emergency obstetric and newborn care service delivery point. Across settings, three facilities in DRC adequately provided selected elements of clinical management of rape. Safe abortion was unavailable. Many providers lacked essential knowledge and skills. Focus groups revealed limited knowledge of available RH services and socio-cultural barriers to accessing them, although participants reported a remarkable increase in use of facility-based delivery services.

**Conclusion:**

Although RH services are being provided, the availability of good quality RH services was inconsistent across settings. Commodity management and security must be prioritized to ensure consistent availability of essential supplies. It is critical to improve the attitudes, managerial and technical capacity of providers to ensure that RH services are delivered respectfully and efficiently. In addition to ensuring systematic implementation of good quality RH services, humanitarian health actors should meaningfully engage crisis-affected communities in RH programming to increase understanding and use of this life-saving care.

## Background

Reproductive health (RH) problems are a leading cause of death and ill-health among women and girls of childbearing age globally [1]. During conflict and natural disasters, access to health services often decreases [2] while RH needs increase [3]. By the end of 2013, an estimated 51.2 million people remained forcibly displaced within their own country or as refugees [4]. As an essential component of humanitarian health response, addressing RH is critical to saving lives and improving the well-being of these crisis-affected populations.

From 2002 to 2004, the Inter-agency Working Group on Reproductive Health in Crises (IAWG) conducted a global evaluation of RH in humanitarian settings [5]. The evaluation included a qualitative study on the availability, quality, and utilization of RH services in three humanitarian settings.^a^ Findings demonstrated that although RH was clearly on the agenda at a policy level, the quality of services was variable. Researchers documented gaps in family planning (FP) services, emergency obstetric and newborn care (EmONC), as well as services for HIV and other sexually transmitted infections (STI). Response to gender-based violence (GBV) was the weakest area assessed, and internally displaced persons (IDPs)—as compared to refugees—were found to lack critical access to RH care. Since 2004, some components of RH have been studied in different humanitarian settings, but research remains limited [6].

From 2012 to 2014, IAWG undertook a second global evaluation of RH in humanitarian settings. The present article describes one component of this project: a cross-sectional, mixed methods case study on RH services for conflict-affected communities in Burkina Faso, Democratic Republic of the Congo (DRC), and South Sudan. The purpose of the study was to document the current availability, quality, utilization of, and access barriers to RH services in selected humanitarian settings in order to contribute to the evidence base informing humanitarian health-related policy and programming.

## Methods

### Study setting

The assessment sites included: the Seno, Soum, and Oudalan provinces of the Sahel Administrative Region in Burkina Faso, the Masisi Health Zone in North Kivu Province in DRC, and Maban County in South Sudan.^b^ Countries met at least three of the following criteria: defined as low income by the World Bank classification in 2012; classified as “Warning” in the Failed States Index; experienced conflict during 2010-2012 per the Uppsala University Conflict Database; defined as “Stressed,” “Crisis” or “Emergency” on the Famine Early Warning System; or had experienced a major natural disaster that warranted the launch of a flash appeal during 2011 or 2012. Within each country, site selection criteria included a robust humanitarian health response and accessibility.

Although the study settings are all in sub-Saharan Africa, they reflect different types of humanitarian crises. The Sahel Region in Burkina Faso represents a more traditional displaced setting in which the majority of refugees (from Mali) reside in three UNHCR-managed camps in an impoverished yet stable rural setting [7]. In volatile North Kivu, however, only 6% of the IDPs live in camps [8]; both IDPs and host communities are affected by ongoing conflict. Maban County in South Sudan is a mixture of the two: the majority of refugees fleeing fighting in Sudan reside in four UNHCR-managed camps, and the host community itself is fragile with exceptionally poor infrastructure and episodic violence [9].

### Study design

This cross-sectional, mixed methods case study improved on the 2004 evaluation by employing both a quantitative approach that included assessments of health facilities purposively selected from those providing services to crisis-affected populations as well as an assessment of a convenience sample of providers’ knowledge and attitudes, and a qualitative approach using focus group discussions (FGDs). The goal of the quantitative component was to document the availability, quality, and utilization of RH service provision in the three settings. The purpose of the qualitative component was to explore access barriers.

Data collection tools were adapted from existing tools by an IAWG working group and translated into French. Ethical approval was obtained through the Columbia University Institutional Review Board as well as the Ministries of Health (MOH) in each setting. Informed verbal consent was obtained from all respondents.

#### Quantitative component

The quantitative component assessed health facilities with regards to availability, quality, and utilization of FP services, EmONC, comprehensive abortion care, as well as key aspects of clinical management of rape (CMoR), and HIV and other STI services. Data on general infrastructure, financial support, and human resources were also collected. The facility assessments used interviews with key staff, clinical register review, and room-by-room inventory of essential supplies and equipment. A facility was designated as capable of providing the specific service based on the following criteria: services were provided in the preceding three months (as self-reported by providers), skilled staff were in place (a mid-level provider or doctor who self-reported training received), and minimum essential equipment and supplies (described in the respective sections) necessary to provide the services were in evidence on the day of the assessment. The specific criteria for each service are detailed in the appendices.

If a service had not been provided in the three prior months, facility staff were asked to identify the primary reason from one of three categories: 1) lack of staff or untrained staff; 2) lack of supplies, equipment, or drugs; or 3) lack of authorization (by MOH or facility director) to provide the service. Utilization of services was measured via service statistics from the six previous months. However, many of these data were missing due to poor registers or the absence of key data points and were therefore excluded from this paper.

In addition, self-completed close-ended questionnaires were used to assess providers’ knowledge and attitudes about RH service provision to help determine quality of care. These findings are described in the text whereas the data from the facility assessments are presented in the tables.

#### Qualitative component

The qualitative component helped to identify barriers to accessing care. The purpose of the FGDs with community members was to gather data on attitudes related to RH, knowledge of existing services, and challenges to accessing RH services.

### Data collection and analysis

The assessments took place between July and October 2013. Data collectors participated in a two-day training prior to data collection.

For the quantitative data collection, a list of all available health facilities serving both host and displaced populations (health center-level and above) was generated in each setting with the assistance of the respective MOH and UNHCR country offices. Facilities inaccessible due to insecurity and physical barriers, such as poor roads, were excluded. All remaining facilities (63) were assessed in each setting (Table 1). A convenience sample of 42 providers at a selection of facilities completed a questionnaire to assess knowledge and attitudes.

**Table 1:** Data collection by method

	Facility assessments	Provider assessment	FGD: No. unmarried women	FGD: No. married women	FGD: No. unmarried men	FGD: No. married men
**Burkina Faso**	28	11	21	20	16	20

**DRC**	26	13	29	38	28	38

**South Sudan**	9	18	20	31	21	20

**Total**	**63**	**42**	**70**	**89**	**65**	**78**

For the qualitative component, a total of 34 FGDs were held with 273 displaced persons and 29 members of the host communities in groups of married women, married men, unmarried women and unmarried men. Consecutive translation was used; multiple facilitators took notes to enhance triangulation (Table 1).

Quantitative data were entered into CS Pro version 5.0 and analyzed using SPSS version 21 (IBM Corp., Armonk, NY, USA); qualitative data were analyzed using thematic analysis [10].

## Findings

For each technical component of RH, we first describe the facility assessment findings, including provider knowledge and attitudes. We then describe the perceptions and responses from the FGDs.

### General infrastructure

General infrastructure of health facilities was assessed in terms of functioning power and water supplies, supplies for minimum infection prevention, as well as the availability of at least one provider at night and on the weekends (Table 2). Most of the hospitals had these elements in place whereas availability at health centers varied. Supplies for minimum infection prevention were inconsistent across settings: two out of four hospitals assessed and 50% or fewer of the health centers had all supplies in evidence at the time of the assessment (Additional file 1: Appendix A). Few facilities (none in DRC; two in South Sudan, and ten in Burkina Faso) had at least one provider trained to provide adolescent-friendly RH services.

**Table 2:** General infrastructure (n=63 health facilities)

	Mean catchment population^1^	Mean number of beds	At least 1 qualified health provider available 24/7	Functioning power supply	Functioning water supply	Minimum infection prevention supplies^2^
**Burkina Faso (n=28)^3^**						

Hospital (n=3)	608,320	89	2 (66.7%)	3 (100%)	3 (100%)	1 ND* (1)

Camp health center (n=4)	18,452	6	3 (100%) ND* (1)	2 (66.7%) ND* (1)	4 (100%)	0 ND* (1)

Non-camp health center (n=21)	6,782	10	13 (61.9%)	13 (76.5%) ND* (4)	16 (80%) ND* (1)	5 (23.8%)

**DRC (n=26)^4^**						

Hospital (n=1)	378,000	171	1	1	1	1

Health center (n=25)	12,870	8	18 (75%)	10 (40%)	14 (56%)	4 (16%)

**South Sudan (n=9)^5^**						

Hospital (n=1)	209,700	60	ND*	1	1	0

Health center (n=8)	ND*	16 (range 2-67)	1 (12.5%)	4 (50%)	8 (100%)	4 (50%)

*ND = no data

^1^ Mean catchment population includes both host and displaced populations with the exception of the camp health facilities in Burkina Faso which served primarily refugees.

^2^ See Additional file 1: Appendix A for details on minimum infection prevention supplies.

^3^ The MOH manages three hospitals and 21 non-camp health centers while the four camp health centers are NGO-managed. The non-camp health centers primarily serve the host community whereas the camp facilities serve refugees. The hospitals serve both populations.

^4^ All facilities from DRC are MOH-managed, but the hospital and 15 health centers received some NGO support for health.

^5^ The hospital and one health center are MOH-managed, six health centers are NGO-managed, and one health center is managed by a religious mission.

Despite this, focus groups with refugees residing in camps in Burkina Faso and South Sudan reported satisfaction with the health services. They commented that facilities were within 30 minutes walking distance or transport was available for emergencies. In DRC, however, focus group participants provided mixed feedback including some complaints about the quality of care, such as clinic staff privileging people they know and stock-outs of medicine and supplies. Access was variable; non-camp focus groups reported the longest distance to a health facility, with one group reporting that the nearest health center was two to three days’ walk. Focus groups with displaced communities in all settings said health care was free of charge.

### Family planning (FP)

Functioning FP service delivery points included the ability to adequately provide a minimum method mix: intra-uterine device (IUD), implant, oral contraceptive pill (OCP), and injectable. Data on permanent FP methods and emergency contraception (EC) after unprotected sex were also collected. Functioning FP service delivery points were limited in Burkina Faso and DRC and nonexistent in South Sudan (Table 3).

**Table 3:** Functioning family planning (FP) service delivery point (n=63)

	Oral contraceptive pill (OCP)	Injectable contraceptive	IUD	Implant	Functioning FP service delivery point^1^
**Burkina Faso (n=28)**					

Hospital (n=3)	3 (100%)	3 (100%)	3 (100%)	3 (100%)	**3 (100%)**

Camp health center (n=4)	3 (100%) ND* (1)	3 (100%) ND* (1)	1 (25%)	1 (25%)	**1 (25%)**

Non-camp health center (n=21)	17 (81%)	17 (81%)	1 (4.8%)	8 (40%) ND* (1)	**1 (4.8%)**

**DRC (n=26)**					

Hospital (n=1)	1	1	1	1	**1**

Health center (n=25)	12 (48%)	10 (40%)	9 (36%)	5 (20%)	**5 (20%)**

**South Sudan (n=9)**					

Hospital (n=1)	0	ID**	0	0	**0**

Health center (n=8)	1 (12.5%)	1 (12.5%)	0	0	**0**

*ND = no data

**ID = incomplete data. The hospital met all the indicators but data on availability of injectables at the time of the assessment were missing.

^1^ Defined as a facility able to provide IUDs, implants, OCPs, and injectables. A facility was classified as able to provide each method if the following criteria were met: self-reported provision of the service in the previous 3 months, at least one provider trained in FP service provision, and presence of minimum essential supplies and equipment on the day of the assessment. See Additional file 2: Appendix B for details.

In South Sudan, five facilities reported providing OCPs and four reported providing injectables in the previous three months, yet only one met the criteria to adequately provide these contraceptives; lack of supplies at the time of the assessment was the primary reason for facilities failing to meet the criteria (Additional file 2: Appendix B). Permanent and long-acting methods were not available at any facilities, although one health center reported having provided implants in the previous three months. Providers cited all possible reasons: lack of authorization, supplies, and trained staff. Three facilities reported providing EC in the previous three months; scarce supplies and lack of authorization were the primary reasons given for not providing EC. Questionnaires revealed that some providers felt personal discomfort with FP services and had personal beliefs that may have influenced their professional conduct.

In Burkina Faso and DRC, the four hospitals met the requirements for a functioning FP delivery point (Table 3). Among health centers in both settings, short-acting methods were more available than long-acting. In Burkina Faso, all camp facilities and 81% of non-camp health centers sufficiently provided short-acting methods, yet only one of each met the criteria to provide IUDs and less than half adequately provided implants (Additional file 2: Appendix B). Among health centers in DRC, coverage was variable with 48% adequately providing OCPs and less than half meeting the criteria to provide injectables; 36% and 20% adequately provided IUDs and implants, respectively. Insufficient supplies were a significant barrier in both settings. For example, although all camp facilities and 90% of non-camp facilities in Burkina Faso reported providing implants in the previous three months, only one and 40%, respectively, had the minimum essential supplies at the time of the assessment, mainly due to lack of forceps. In DRC, 72% of health centers reported providing injectables in the three months prior but only 54% had injectables in evidence at the time of the assessment. Lack of trained staff was also a challenge to providing FP in DRC, while the large majority of facilities in Burkina Faso had at least one staff trained in each method. Some permanent methods were available: the hospital in DRC reported having performed tubal ligation and one hospital in Burkina Faso performed vasectomy in the previous three months. Emergency contraception had reportedly been provided at 42% and 36% of facilities in DRC and Burkina Faso, respectively, in the three months prior. Questionnaires found that some providers in all three settings maintained negative attitudes toward women using contraception without their husbands’ consent (Additional file 8: Appendix H).

FGDs in all settings revealed significant socio-cultural barriers and misconceptions regarding FP. Participants reported that large families were socially valued, and contraception was associated with sex work or sex outside of marriage, which were viewed negatively. Further, awareness of available services was limited in all settings. In DRC, some women reported that they were required to present an authorization letter or be accompanied by their husband to access FP services, although no such policy was in place. Participants in Burkina Faso and DRC said that unmarried and adolescent women had the most difficulty accessing FP services, while in South Sudan, unmarried women were generally unaware of family planning methods.

### Emergency obstetric and newborn care (EmONC)

A functioning basic or comprehensive EmONC service delivery point was defined as being able to provide the applicable signal functions,^c^ or life-saving obstetric interventions, as recommended by WHO [11]; availability of partographs, blood pressure cuff, and stethoscope, which are essential to provide good delivery care, were also required. In general, health centers should provide basic EmONC (BEmONC) and referral hospitals comprehensive EmONC (CEmONC). Across settings, all hospitals except for one in Burkina Faso (which had not provided assisted vaginal delivery) reported providing all elements of CEmONC in the previous three months. Yet only one hospital in Burkina Faso met the criteria for a functioning CEmONC delivery point when supplies and equipment were assessed (Table 4). Of the health centers, only one in South Sudan could be defined as a functioning BEmONC delivery point. In DRC, the only adequately functioning BEmONC service delivery point was the hospital. Per WHO guidance, the minimum acceptable level of coverage is five functioning health facilities providing EmONC, at least one of which provides CEmONC, per 500,000 population [11]. None of the settings met this benchmark for minimum BEmONC or CEmONC coverage, although BEmONC coverage in South Sudan was unknown because the population was too transient to establish a reliable mean health center catchment population. Facilities located in camps reported functioning referral systems for obstetric emergencies; however, non-camp facilities reported weaker or non-functional referral mechanisms.

**Table 4:** Functioning EmONC and post-abortion care (PAC) delivery points, additional elements of newborn care, and induced abortion (n=63)

	Functioning BEmONC service delivery point^1^	Functioning CEmONC service delivery point^1^	Essential elements of newborn care^2^	Functioning PAC service delivery point^3^	Induced abortion^4^
**Burkina Faso (n=28)**					

Hospital (n=3)	1 (33.3%)	1 (33.3%)	2 (66.7%)	3 (100%)	0**

Camp health center (n=4)	0	NA	1 (25%)	1 (25%)	0

Non-camp health center (n=21)	0	NA	2 (9.5%)	0	0

**DRC (n=26)**					

Hospital (n=1)	1	0*	1	1	0**

Health center (n=25)	0	NA	0	11 (44%)	0**

**South Sudan (n=9)**					

Hospital (n=1)	0	0	0	1	0

Health center (n=8)	1 ND (1)	NA	2 (25%)	1	0

* Minimum criteria for all CEmONC signal functions met except for blood transfusion

**Health facility assessments found that none of the facilities provided induced abortion. However, some providers reported that they had performed induced abortion in the previous three months.

^1^ Defined as a facility able to provide all nine (comprehensive) or seven (basic) EmONC signal functions. A facility was classified as able to provide each signal function if the following criteria were met: self-reported provision of EmONC services in the previous three months, at least one provider trained in basic or CEmONC, presence of minimum essential supplies and equipment for each signal function on the day of the assessment. See Additional file 3: Appendix C for details. Hospitals that met the criteria for a CEmONC facility are not included in the BEmONC data.

^2^ Defined as having at least one skilled staff trained to provide neonatal resuscitation, breastfeeding support, newborn infection management, thermal care, cord care, kangaroo care, delivery practices for PMTCT and presence of minimum essential equipment and supplies for neonatal resuscitation and infection management. See Additional file 4: Appendix D for details.

^3^ Defined as having provided PAC services in the previous three months (self-reported), offering FP to all PAC clients, presence of minimum essential equipment and supplies for PAC using MVA or misoprostol. See Additional file 5: Appendix E for details.

^4^ Self-reported provision of the service in the previous three months

Across settings, assisted vaginal delivery was particularly limited: of the health centers, one in DRC, three in South Sudan, and none in Burkina Faso were able to adequately provide this signal function, primarily due to lack of authorization and absence of supplies (Additional file 3: Appendix C).

In Burkina Faso, the large majority of facilities had at least one provider trained to provide BEmONC. Parenteral drugs were more available at non-camp health centers than camp centers, although the availability of removal of retained products was minimal at both with only one of each adequately providing this signal function. In DRC, CEmONC was not fully available and adequate BEmONC was extremely limited: of the 26 health facilities, the hospital was the only adequately functioning EmONC delivery point, and it only met the criteria as a BEmONC facility due to missing supplies for blood transfusion. In South Sudan, most health centers sufficiently provided manual removal of the placenta yet only two adequately provided parenteral anticonvulsants; the referral hospital lacked elements to sufficiently provide assisted vaginal delivery and did not have partographs. Providers across settings reported a dearth of equipment and drugs as the primary barrier to providing adequate basic and CEmONC followed by shortages of trained staff; some providers also reported that their facility lacked authorization to provide certain signal functions.

Data on additional essential elements of newborn care were also collected, including having provided neonatal resuscitation in the previous three months, the availability of skilled staff trained to provide breastfeeding support, newborn infection management, thermal care, cord care, kangaroo care, delivery practices for prevention of mother-to-child transmission of HIV (PMTCT), as well as drugs for infection management. Availability of adequate newborn care was limited across settings (Table 4). Of the health centers that failed to provide adequate neonatal resuscitation in Burkina Faso and DRC, the majority reported lack of supplies as the main reason for not providing the service: two-thirds did not have a resuscitation bag and infant face mask in DRC and 80% lacked corticosteroids in Burkina Faso (Additional file 4: Appendix D). In addition, providers in many facilities across settings lacked training in newborn infection management.

Provider questionnaires revealed varied knowledge of EmONC. For example, on average across settings, providers could name most of the nine key observations for labor monitoring but fewer than half of the eight essential activities to manage post-partum hemorrhage (Additional file 8: Appendix H).

Despite the significant gaps in good quality EmONC services, focus groups and community leaders reported positive experiences with maternal health services. Notably, all groups—including men—in the three settings were aware of the advantages of women delivering in a health facility. Remarkably, whereas childbirth had previously occurred at home with a traditional birth attendant, respondents reported that facility births had now become a norm. They attributed their attitude and behavior changes to education campaigns and outreach by health providers. In all settings refugees and IDPs said that they were aware of the existing maternal health services and that care was free. One FGD in DRC reported that distance impeded access to delivery care.

### Comprehensive abortion care

Comprehensive abortion care included the provision of post-abortion care (PAC) as well as safe induced abortion according to national law [12]. Of the five hospitals assessed, all met the requirements for a functioning PAC service delivery point, defined as being able to adequately provide manual vacuum aspiration (MVA) or misoprostol and offering at least one FP method to post-abortion clients (Table 4). Among health centers, almost half in DRC met the criteria to adequately provide PAC. Fewer health centers in Burkina Faso and South Sudan sufficiently provided PAC, with providers reporting they were unauthorized to do so. Providers in all settings also cited scarce supplies followed by dearth of trained staff as barriers to service provision. MVA was the most common means of uterine evacuation; and two facilities in Burkina Faso and three in both DRC and South Sudan had misoprostol for PAC available at the time of the assessment (Additional file 5: Appendix E.)

Although induced abortion is legally permitted under certain circumstances^d^ in all settings [13], the facility assessments found that none of the facilities provided this service. However, interviews with providers and the questionnaires suggested that abortion may be available at some health facilities in DRC and Burkina Faso, although this remains unclear. Providers in all settings reported that their facility lacked authorization to perform induced abortions and few staff were trained.

Across settings, FGDs revealed negative attitudes toward abortion, which they said conflicted with religious beliefs. However, all reported that some women and girls in their communities resorted to unsafe abortion.

### Clinical management of rape (CMoR)

While the minimum package of CMoR for low-resource settings includes 25 elements [14], selected key elements of CMoR were assessed, including the availability of EC, post-exposure prophylaxis for HIV (PEP), and antibiotics for STI prevention, the provision of these drugs in the previous three months, as well at least one staff trained to provide CMoR.

Across settings, only three facilities, all in DRC, had these selected elements in place (Table 5). Some availability of this care in DRC—as opposed to the other two settings—was not surprising given the international attention to the widespread sexual violence in DRC’s conflict-affected areas [15]. Yet three of 26 facilities still constituted limited coverage, and it was unclear whether these three facilities provided all components of the minimum package of CMoR [14]. A paucity of drugs was the primary barrier reported in DRC. For instance, more than 80% of facilities reported providing presumptive STI treatment for CMoR in the previous three months, but only 8% had all necessary antibiotics for STIs available on the day of the assessment (Additional file 6: Appendix F).

**Table 5:** Facilities with essential drugs and at least 1 qualified staff to provide clinical management of rape (CMoR) (n=63)

	At least 1 provider qualified to provide CMoR	Post-exposure prophylaxis (PEP)^1^	Emergency contraception (EC)^1^	Antibiotics to prevent STI^1^	Facilities with essential drugs and ≥1 qualified staff for CMoR
**Burkina Faso (n=28)**					

Hospital (n=3)	2 (66.7%)	2 (66.7%)	0	2 (66.7%)	**0**

Camp health center (n=4)	1 (33.3%) ND* (1)	0	2 (67.7%) ND* (1)	2 (50%)	**0**

Non-camp health center (n=21)	10 (47.6%)	0	6 (28.6%) ND* (1)	9 (42.9%)	**0**

**DRC (n=26)**					

Hospital (n=1)	1	1	1	1	**1**

Health center (n=25)	18 (72%)	10 (40%)	12 (48%)	2 (8%)	**2 (8%)**

**South Sudan (n=9)**					

Hospital (n=1)	0	0	0	0	**0**

Health center (n=8)	1 (25%) ND* (4)	1 (14.3%) ND* (1)	1 (14.3%) ND* (1)	2 (28.6%) ND* (1)	**0** ND* (2)

*ND = no data

^1^ Self-reported provision of the service in the previous three months and presence of supplies on the day of the assessment. See Additional file 6: Appendix F for details.

A dearth of supplies was found in the other two settings as well. In Burkina Faso, all four camp facilities reported having provided EC and antibiotics for STIs as part of CMoR in the previous three months; three of the four reported providing PEP. In South Sudan, two health centers reported providing all drugs—EC, PEP, and antibiotics for STIs—for CMoR in the prior three months. Yet, at the time of the assessment, all lacked supplies. In Burkina Faso, PEP was not available at any health centers; of the health centers that did not provide PEP in the previous three months, half reported that they were not authorized to do so.

Regarding staff, almost three quarters of health facilities in DRC had at least one provider trained in CMoR. Trained providers were less available in Burkina Faso and South Sudan. Questionnaires demonstrated that, on average, providers across settings could identify fewer than half of the eleven key CMoR activities (Additional file 8: Appendix H).

Responses from FGD participants about sexual violence varied. Participants in DRC were the most knowledgeable about where to seek services and were generally aware of the importance of seeking care within 72 hours after rape. Informants in South Sudan and Burkina Faso were unaware of existing services or reasons to seek health care. However, across settings, the FGDs reported that most rape survivors would not come forward due to fears of stigma and rejection as well as concerns about confidentiality. All said that young unmarried women were at risk of sexual assault. Women in DRC reported that sexual violence was widespread, including at home and by armed groups. Women in Burkina Faso and South Sudan described marital rape as commonplace; they reported that sexual violence outside of marriage did occur, primarily perpetrated by members of the refugee or host communities, but was rare.

### HIV and other STIs

Adequate provision of STI services (syndromic or laboratory testing and treatment) and PMTCT included self-reported provision of the service in the preceding three months and the availability of essential drugs on the day of the assessment. Data were collected on self-reported provision of anti-retroviral therapy (ART) for people living with HIV (PLHIV), voluntary counseling and testing for HIV (VCT), and condoms in the previous three months.

Adequate STI and HIV services were available at the hospital in DRC and two of three hospitals in Burkina Faso, but nonexistent at the hospital in South Sudan (Table 6). Most facilities across settings reported having provided STI care in the previous three months, yet, apart from non-camp facilities in Burkina Faso, the majority failed to meet the criteria to adequately provide these services due to lack of antibiotics (Additional file 7: Appendix G). In South Sudan, ARVs for PMTCT and ART had not been provided at any health facility in the previous three months; six of the nine health facilities reported that they lacked authorization to administer ARVs and others reported they lacked supplies. In DRC, none of the health centers adequately provided PMTCT due to lack of supplies as well as lack of trained staff. In Burkina Faso, most services were available in non-camp facilities; however, ART outside of the hospitals was rarely available, primarily due to policy barriers, with 18 of the 25 health centers reporting they were not authorized to provide ART to PLHIV. Questionnaires revealed that, on average across settings, providers could name half of five key elements of care for someone presenting with symptoms of an STI (Additional file 8: Appendix H).

**Table 6:** HIV and other sexually transmitted infection (STI) services (n=63)

	**Syndromic or laboratory diagnosis and treatment of STIs**^1^	ARVs for HIV+ mothers and newborns in maternity^1^	ART for people living with HIV^2^	Voluntary HIV counseling and testing^2^	Condom provision^2^
**Burkina Faso (n=28)**					

Hospital (n=3)	2 (67%)	3 (100%)	3 (100%)	3 (100%)	3 (100%)

Camp health center (n=4)	1 (25%)	1 (25%)	1 (25%)	4 (100%)	3 (75%)

Non-camp health center (n=21)	21 (100%)	18 (90%) ND* (1)	3 (14.3%)	19 (90.5%)	18 (85.7%)

**DRC (n=26)**					

Hospital (n=1)	1	1	1	1	1

Health center (n=25)	2 (9%) ND (2)	0	1 (4%)	6 (25%)	12 (48%)

**South Sudan (n=9)**					

Hospital (n=1)	0	0	0	0	0

Health center (n=8)	3 (38%)	0	0	1 (12.5%)	4 (50%)

*ND = no data

^1^ Self-reported provision of the service in the previous three months and presence of essential equipment and supplies on the day of the assessment. See Additional file 7: Appendix G for details.

^2^ Self-reported provision of the service in the previous three months

Most FGD participants had heard about HIV yet stigma and misconceptions abounded. Condom knowledge and use, which focus group participants often associated with sex workers, was low. Although condoms were provided at roughly half of the health facilities in South Sudan and DRC and more than three quarters in Burkina Faso, only in Burkina Faso were the majority of FGD participants aware of their availability. In all sites, young unmarried women were the least knowledgeable of HIV, STIs, and ways to minimize transmission; some young women in DRC had never heard of HIV or AIDS. Participants in Burkina Faso and DRC cited lack of confidentiality as a key barrier to seeking HIV/AIDS services.

## Discussion

A decade has passed since the availability, quality, and barriers to RH services have been assessed across crisis-affected settings. Although poor data quality prevented analysis of utilization data, the mixed methods approach allowed us to estimate quality of care through the availability of a minimum standard of RH services and provider knowledge and attitudes, as well as understand access barriers.

The assessment criteria held facilities to a strict minimum standard of care, based on international guidance, requiring them to have provided the service recently as well as have trained staff and specific essential supplies in place. Findings revealed a striking inconsistency between the self-reported provision of RH services and the availability of the minimum supplies and trained staff to adequately provide them: many facilities reported having recently provided a number of RH services, yet—apart from the hospitals in DRC and Burkina Faso—the availability of a minimum standard of quality RH services was generally limited. A service cannot be considered available when minimum essential elements to provide the service are not present or face regular stock-outs. Further, many providers lacked critical RH knowledge and some exhibited biases that weakened good quality care.

Compared to findings from other research in humanitarian settings, [5,6,16] positive developments were evident in all settings: some health facilities are meeting minimum standards. Among those that did not meet minimum standards for specific RH components, all facilities reported recent, albeit inadequate, provision of some RH services, indicating RH programming is being implemented but facilities need assistance to meet standards. Low-income settings with weak or limited RH services before the crisis can benefit from humanitarian interventions. The three countries for this study have consistently ranked low on the Human Development Index: South Sudan (as part of Sudan) was ranked 171 out of 186 in 2013, Burkina Faso was 183, and DRC tied for last [17]. Their weak health systems suggest that many RH services are not available to the general population, and humanitarian agencies have contributed to decentralizing services to rural areas that may otherwise take decades to receive such support. For example, an assessment in North Kivu in 2002 found that condoms were generally not available at health facilities and only one facility assessed offered VCT for a fee [18]. Now the hospital and some health facilities in the Masisi Health Zone provide free VCT and condoms. Further, displaced communities in all sites reported significant changes in delivery practices: previously women had given birth at home whereas they now sought facility-based care. These positive behavioral developments resulted largely from outreach by humanitarian actors.

The study also found a number of critical gaps in service provision, which are particularly worrying when situated against the backdrop of the RH needs in the three settings. Only three of the 25 health centers assessed in DRC provided all assessed elements of CMoR, despite extensive sexual violence documented in the area [19-22]. In South Sudan, 2,054 women die of pregnancy-related causes per 100,000 live births and have an average of 6.7 children, among the worst maternal mortality and highest fertility rates in the world [23]. The South Sudan county hospital—among other serious gaps—did not meet the criteria for even a basic EmONC facility nor did it offer any FP methods, including condoms. Misconceptions and cultural barriers regarding FP were widespread, and many providers avoided discussing the topic with clients. In sub-Saharan Africa generally, an estimated 97% of abortions are unsafe [24]. Concurrently, findings show that safe abortion was not systematically available in any assessment sites, and availability of PAC and a full package of FP services were limited in health centers. This deadly combination of high RH needs, limited and poor quality care, lack of knowledge, and cultural barriers that thwart health-seeking behavior demands urgent attention.

The findings highlight that, in addition to expanding RH service provision, attention is needed to ensure services are of good quality and meet minimum standards; socio-cultural access barriers to all RH components also need addressing. The poor availability of utilization data (e.g., service statistics) underscores the importance of using data to improve services. When services are introduced, attention must be paid to ensure that key data are collected in facility registers so staff can monitor progress. Among other recommendations outlined below, health actors must prioritize and support RH programming and ensure RH is integrated into their primary health care activities.

### Community engagement

Findings from FGDs highlighted the importance of community engagement. Across settings, many refugees and IDPs reported being unaware of existing RH services or lacking information as to why they should seek care. For example, many FGD participants did not know that medicine to decrease risk of pregnancy or HIV transmission after rape or intercourse existed. Even those who knew about the existing services—and the importance of accessing care—disclosed that they were unlikely to seek care at a health facility. Cultural norms, such as the relationship between social status and number of children, as well as social sanctions against PLHIV, rape survivors, and women who use FP methods undermined health-seeking behavior. Some FGDs also expressed concerns about confidentiality and the quality of existing RH services.

Some providers did not recognize the need to expand specific RH services, and only provided care—particularly FP methods—to clients who specifically requested it. Low use of RH services does not reflect a true lack of demand but indicates a need for education and engagement as well as integration of RH with other health services. Provider training and awareness-raising of service availability are good first steps but insufficient. Meaningful community participation and engagement, grounded in a rights-based approach and evidence-informed programming, are necessary to increase access to and use of RH services. Indeed, the beneficial changes in community norms and behaviors regarding facility-based delivery resulted from systematic outreach by health actors. A small yet robust body of evidence suggests that community participation in primary health care is associated with increased utilization as well as improved health outcomes [25]. These findings provide support that community engagement is not just desirable but essential for successful RH service implementation.

### Commodity security

Poor commodity security and supply chain management obstructed good quality service delivery in all settings. Providers overwhelmingly reported a paucity of drugs as the primary barrier to providing adequate RH care. Further, many facilities reported providing RH care yet lacked sufficient equipment and supplies to adequately do so. Action to address commodity security and management is urgently needed. As a starting point, a comprehensive logistical audit, including evaluation of policy and protocols, budgetary constraints, forecast accuracy, storage conditions, and staff capacity would benefit RH provision in the three settings. Capacity development of national and international staff at every point in the supply chain as well as improved logistics management information systems can help strengthen the delivery system. Where feasible, respective MOHs should establish or strengthen contingency stocks of RH supplies to prevent stock outs. Evidence-based advocacy may be required to integrate RH commodity security into national policies and programs. Finally, sustained funding is necessary to realize these recommendations. The establishment of a functioning commodity management system is essential to ensure consistent access to care.

### Capacity development

Gaps in RH care resulted from a dearth of skilled staff as well. Primary training gaps included long-acting and permanent FP methods, newborn infection management, adolescent-friendly services, induced abortion, and assisted vaginal delivery. Few staff in Burkina Faso and South Sudan had training in CMoR. Further, questionnaires revealed that, even when trained providers were in place, many lacked essential knowledge and skills. An effective, good quality humanitarian health response requires skilled staff with an up-to-date knowledge and skills base. Health staff providing RH services need competency-based clinical trainings on RH as well as health systems broadly. Shortcomings in health service provision result not only from weak clinical competence but also from lack of non-technical skills such as poor situation awareness, decision-making, and inter-personal skills including communication and teamwork. These social and cognitive skills can increase patient safety and streamline service delivery; moreover, these skills can be taught and learned [26]. Clinical trainings should integrate these important elements as well as reinforce humanitarian principles, professional ethics, and accountability to affected communities—cornerstones of good quality health care. Supportive supervision should be practiced to help providers improve and maintain these skills and address gaps in service provision.

### Policy

Restrictive national policies as well as providers’ lack of knowledge of supportive policies and protocols undermined RH service provision. Providers at health centers reported that lack of authorization significantly restricted the provision of assisted vaginal delivery, CMoR, and ART, especially in Burkina Faso. Mid-level providers should be allowed to provide many RH services, such as all elements of BEmONC, to expand service availability at the health center level [27]. As far as we could determine, health centers in the three countries are mandated to provide all assessed RH services except for surgery, blood transfusion, safe abortion, and in some cases initiation of ART, suggesting incorrect knowledge of MOH policies by those who cited a lack of authorization for many services.

Safe abortion was an alarming gap across all facilities. In the three countries, abortion is legally permitted when the woman’s life is at risk; Burkina Faso has additional legal indications for abortion [13]. Yet almost all providers reported that abortion was unauthorized in their facility. Although international guidance on RH in emergencies includes safe abortion to the extent of the law [12,28,29], abortion has largely been ignored by humanitarian health actors [30,31]. An estimated 13% of all maternal deaths are caused by unsafe abortion globally; more than 98% occur in the world’s poorest countries, where the majority of humanitarian emergencies occur [32]. Respective MOHs as well as international humanitarian health actors must prioritize comprehensive abortion care in crisis-affected settings as well as identify and address restrictive policies and discrepancies between policy and practice.

### Adolescent RH

Across settings, few facilities had at least one provider trained to provide adolescent-friendly RH services. Focus groups revealed that young unmarried women were least knowledgeable about HIV, STIs, and condom use compared to unmarried men, married men, and married women. They were the least likely to seek FP services, and FGDs reported that young women were among the most vulnerable to sexual assault. Communities expressed fears that making contraceptives available to adolescents would increase sexual activity outside of marriage.

Adolescents in developing countries are more likely to marry younger, resort to unsafe abortion, and die in childbirth than their counterparts in wealthier nations [33]. Adolescents in crisis-affected settings have additional vulnerabilities, risks, and needs [34]. In North Kivu, for example, only 9% of women and girls aged 15-24 reported using a condom the last time they had sexual intercourse with a casual partner [35]. According to a review of adolescent RH programs in humanitarian settings, successful programs ensure adolescent participation in programming, work to build community trust and adult support, and secure qualified and dedicated staff [34]. Findings demonstrate need for adolescent-specific interventions in the three settings.

## Limitations

This study faced a number of limitations. Due to missing and poor quality service statistics, utilization could not be assessed. Insecurity and physical obstacles, such as poor roads and rain, were significant barriers across settings and prevented visits to some health facilities. Time pressures, high workloads, and coordination challenges among assessment team members resulted in missing data. Translation error was a possibility, particularly in South Sudan and Burkina Faso where the responses had to be translated from a local language to Arabic or French and then to English (in South Sudan). The respective assessment teams addressed the translation challenges through daily debriefings and group discussions to clarify findings.

## Conclusion

Access to RH services, even in the midst of war or natural disaster, is a human right that saves lives, preserves health, and can enhance physical and mental well-being. Despite the many obstacles to service delivery, communities affected by crises deserve high quality RH care. Progress in advancing and improving the quality of RH in emergencies has been made at the global level in terms of policies, guidelines, and funding [3,36,37]. While it is promising that many health facilities are providing some RH services, there remains an urgent need to address gaps in implementation—in particular safe abortion services—as well as the quality of care, utilization of RH services, and monitoring and evaluation. Minimum quality standards must be met to meet the health needs of affected populations. Yet, only expanding RH service availability is not sufficient. Gaps in management and knowledge, as well as the biases of some providers continue to impede the provision of RH services in humanitarian settings. Though these may be less quantifiable investments for donors and policy makers, merely providing supplies will not result in necessary quality improvements in RH service delivery.

Further, the remarkable changes in health-seeking behavior for pregnancy care warrant further exploration. Indeed, the identification of effective strategies for increasing demand for and use of skilled attendants is at the forefront of the humanitarian neonatal research agenda [38]. Behavior change to increase use of RH services is possible and necessary; improved integration of different RH services within facilities would capitalize on already strong pregnancy-related health-seeking behavior. In addition to ensuring systematic implementation of good quality RH services, humanitarian health actors should—to the extent possible—meaningfully engage crisis-affected communities, especially adolescents, in RH programming to augment access to this life-saving care.

## List of abbreviations used

AIDS: Acquired immunodeficiency syndrome; ARV: Antiretroviral; ART: Antiretroviral therapy; BEmONC: Basic emergency obstetric and newborn care; CmONC: Comprehensive emergency obstetric and newborn care; CMoR: Clinical management of rape; DRC: Democratic Republic of the Congo; EC: Emergency contraception; EmONC: Emergency obstetric and newborn care; FP: Family planning; FGD: Focus group discussion; GBV: Gender-based violence; HIV: Human immunodeficiency virus; IAWG: Inter-agency Working Group on Reproductive Health in Crises; IDP: Internally displaced person; IV: Intravenous; IUD: Intra-uterine contraceptive device; MOH: Ministry of Health; MVA: Manual vacuum aspiration; NGO: Nongovernmental organization; OCP: Oral contraceptive pill; PLHIV: People living with HIV; PAC: Post-abortion care; PEP: Post-exposure prophylaxis; PMTCT: Prevention of mother-to-child transmission; RH: Reproductive health; STI: Sexually transmitted infection; UN: United Nations; UNHCR: United Nations High Commissioner for Refugees; VCT: Voluntary counseling and testing; WHO: World Health Organization.

## Competing interests

The authors declare that they have no competing interests.

## Authors’ contributions

SEC, NC, and MCG led the study conceptualization and design. SKC conceptualized the paper and was the principle author. SEC provided remote technical assistance during data collection; NC participated in the data collection in DRC. SEC and EEW led the quantitative data analysis; SKC conducted the qualitative data analysis. SEC, EEW, NC, and MCG provided substantial feedback. All authors reviewed and approved the final text.

## Endnotes

^a^ The three countries included in the 2004 IAWG evaluation were Republic of Congo, Uganda, and Yemen.

^b^ The data pertain to the assessment settings only. However, for ease of reading, the countries are referred to throughout the article.

^c^ The basic EmONC signal functions include: 1. administer parenteral antibiotics; 2. administer uterotonic drugs (e.g., parenteral oxytocin); 3. administer parenteral anticonvulsants for pre-eclampsia and eclampsia (e.g., magnesium sulphate); 4. perform manual removal of placenta; 5. perform removal of retained products of conception (e.g., manual vacuum aspiration); 6. perform assisted vaginal delivery (e.g., vacuum extraction); 7. perform neonatal resuscitation (with bag and mask). A comprehensive EmONC facility must provide the above signal functions as well as the following two: 8. perform blood transfusion; and 9. perform surgery (e.g., Caesarean section) [11].

^d^ Abortion is permitted in Burkina Faso, DRC, and South Sudan when the woman’s life is at risk. In Burkina Faso, abortion is also allowed in cases of rape, incest, and fetal impairment [13].

## Supplementary Material

Additional file 1 Appendix A Click here for file

Additional file 2 Appendix B Click here for file

Additional file 3 Appendix C Click here for file

Additional file 4 Appendix D Click here for file

Additional file 5 Appendix E Click here for file

Additional file 6 Appendix F Click here for file

Additional file 7 Appendix G Click here for file

Additional file 8 Appendix H Click here for file

## Declarations

This article has been published as part of *Conflict and Health* Volume 9 Supplement 1, 2015: Taking Stock of Reproductive Health in Humanitarian Settings: 2012-2014 Inter-agency Working Group on Reproductive Health in Crises' Global Review. The full contents of the supplement are available online at http://www.conflictandhealth.com/supplements/9/S1. Funding for this supplement was provided by the MacArthur Foundation.

## References

[B1] UNFPA State of the world population 2005 New York

[B2] Banatvala N Zwi AB Public health and humanitarian interventions: developing the evidence base BMJ 2000 321 101 5 10.1136/bmj.321.7253.101 10884265 PMC1127723

[B3] Austin J Guy S Lee-Jones L McGinn T Schlecht J Reproductive health: a right for refugees and internally displaced persons Reprod Health Matter 2008 16 10 21 10.1016/S0968-8080(08)31351-2 18513603

[B4] UNHCR Global Trends Report 2013 2014 Geneva

[B5] Inter-Agency Working Group on Reproductive Health in Refugee Situations Report of an inter-agency global evaluation of reproductive health services for refugees and internally displaced persons 2004 Geneva

[B6] Cf Casey SE Evaluations of reproductive health programs in humanitarian settings: a systematic review Conflict and Health 2015 9 Suppl 1 S1 25685183 10.1186/1752-1505-9-S1-S1 PMC4328944

[B7] UNHCR country operations profile: Burkina Faso http://www.unhcr.org/cgi-bin/texis/vtx/page?page=49e483de6

[B8] UNOCHA Democratic Republic of Congo: internally displaced people and returnees 2002 http://reliefweb.int/sites/reliefweb.int/files/resources/DRC%20Factsheet%20Population%20Movement%20_english_2%20eme%20trimestre%202013.pdf

[B9] UNHCR Refugees in Upper Nile State fact sheet http://data.unhcr.org/SouthSudan/download.php?id=632

[B10] Guest G MacQueen KM Namey EE Applied thematic analysis 2011 Thousand Oaks: Sage

[B11] WHO UNFPA UNICEF AMDD Monitoring emergency obstetric care: a handbook 2009 Geneva

[B12] Inter-Agency Working Group on Reproductive Health in Crisis Situations Inter-agency field manual on reproductive health in humanitarian situations 2010 Geneva 26203479

[B13] Center for Reproductive Rights World’s abortion laws http://worldabortionlaws.com/

[B14] WHO UNHCR Clinical management of rape survivors: developing protocols for use with refugees and internally displaced persons 2004 Geneva 38

[B15] D'Errico NC Kalala T Nzigire LB Maisha F Malemo Kalisya L ‘You say rape, I say hospitals. But whose voice is louder?’ Health, aid and decision-making in the Democratic Republic of Congo Rev African Polit Economy 2013 40 51 66 10.1080/03056244.2012.761962

[B16] McGinn T Reproductive Health of War-Affected Populations: What Do We Know? International Family Planning Perspectives 2000 26 174 180 10.2307/2648255

[B17] UNDP Human development report 2013. The rise of the south: human progress in a diverse world 2013 New York

[B18] JSI Research & Training Institute Assessment of reproductive health in the Democratic Republic of Congo 2002 Arlington, VA: Reproductive Health for Refugees Consortium

[B19] Peterman A Palermo T Bredenkamp C Estimates and determinants of sexual violence against women in the Democratic Republic of Congo Am J Public Health 2011 101 21566049 10.2105/AJPH.2010.300070 PMC3093289

[B20] Wakabi W Sexual violence increasing in Democratic Republic of Congo Lancet 2008 37 15 16 18183654 10.1016/S0140-6736(08)60051-3

[B21] Steiner B Benner MT Sondorp E Schmitz KP Mesmer U Rosenberger S Sexual violence in the protracted conflict of DRC programming for rape survivors in South Kivu Confl Health 2009 3 1 9 10.1186/1752-1505-3-1 19284879 PMC2667421

[B22] Bartels SA Scott JA Mukwege D Lipton RI VanRooyen MJ Leaning J Research patterns of sexual violence in Eastern Democratic Republic of Congo: reports from survivors presenting to Panzi Hospital in 2006 Confl Health 2010 4 9 10.1186/1752-1505-4-9 20444265 PMC2883538

[B23] Ministry of Health Government of Southern Sudan Sudan household health survey 2006 http://www.southsudanmedicaljournal.com/assets/files/misc/SHHS.pdf

[B24] Sedgh G Singh S Shah IH Åhman E Henshaw SK Bankole A Induced abortion: incidence and trends worldwide from 1995 to 2008 Lancet 2012 379 625 632 10.1016/S0140-6736(11)61786-8 22264435

[B25] Bath J Wakerman J Impact of community participation in primary health care: what is the evidence? Aust J Prim Health 2013 10.1071/PY12164 24176202

[B26] Flin R O’Connor P Crichton M Safety at the sharp end: a guide to non-technical skills 2008 Farnham: Ashgate

[B27] Lassi ZS Cometto G Huicho L Bhutta ZA Quality of care provided by mid-level health workers: systematic review and meta-analysis B World Health Organ 2013 91 824 833I 10.2471/BLT.13.118786 24347706 PMC3853954

[B28] WHO Safe abortion: technical and policy guidance for health systems 2012 Geneva 23700650

[B29] African Union Protocol to the African Charter on Human and Peoples' Rights on the Rights of Woman in Africa 2003 http://www.achpr.org/files/instruments/women-protocol/achpr_instr_proto_women_eng.pdf

[B30] Guy S Meaningful change or business as usual? Reproductive health in humanitarian settings Forced Migr Rev 2013 http://www.fmreview.org/25th-anniversary/guy

[B31] McGinn T Parker R, Sommer M Reducing death and disability from unsafe abortion Routledge Handbook of Global Public Health 2011 New York: Routledge 191 198

[B32] WHO Unsafe abortion: global and regional estimates of the incidence of unsafe abortion and associated mortality in 2003 2007 Fifth Geneva

[B33] WHO Adolescent pregnancy: unmet needs and undone deeds: a review of the literature and programmes 2007 Geneva

[B34] Tanabe M Schlecht J Manohar S Adolescent sexual and reproductive health programs in humanitarian settings: an in-depth look at family planning services 2011 New York: Women's Refugee Commission

[B35] Ministère du Plan and the Institut National de la Statistique UNICEF Democratic Republic of the Congo Multi-Indicator Cluster Survey MICS-2010: monitoring the situation of women and children 2010 http://www.childinfo.org/files/MICS-RDC_2010_Summary_Report_EN.pdf

[B36] Tanabe M Schaus K Rastogi S Krause SK Patel P Tracking Humanitarian Funding for Reproductive Health: A Systematic Analysis of Health and Protection Proposals from 2002-2013 Confl Health 2015 9 Suppl 1 S2 10.1186/1752-1505-9-S1-S2 PMC4331814 25798188

[B37] Patel P Roberts B Guy S Lee-Jones L Conteh L Tracking official development assistance for reproductive health in conflict-affected countries PLoS Medicine 2009 6 e1000090 10.1371/journal.pmed.1000090 19513098 PMC2682761

[B38] Morof DF Kerber K Tomczyk B Lawn J Blanton C Sami S Amsalu R Neonatal survival in complex humanitarian emergencies: setting an evidence-based research agenda Confl Health 2014 8 8 10.1186/1752-1505-8-8 24959198 PMC4057580

