# Supplementary material for: Progress and gaps in reproductive health services in three humanitarian settings: mixed-methods case studies
Source: Confl Health. 2015 Feb 2;9(Suppl 1):S3. doi: 10.1186/1752-1505-9-S1-S3 (PMC4331815; doi:10.1186/1752-1505-9-S1-S3)
Supplement: Additional file 1 — Appendix A [file 1752-1505-9-S1-S3-S1.pdf]

Appendix A: Minimum infection prevention supplies, by country

Table A1. Burkina Faso: facilities with minimum infection prevention supplies available (n=28)

	Hospital (n=3)	Camp health center (n=4)	Non-camp health center (n=21)
Washing station with soap	3 (100%)	3 (100%)<br>ND* (1)	21 (100%)
Plastic sheeting	1 (50%)<br>ND* (1)	0<br>ND* (1)	8 (38.1%)
Non-sterile gloves	3 (100%)	4 (100%)	21 (100%)
Sterile gloves	3 (100%)	4 (100%)	21 (100%)
Antiseptics	3 (100%)	4 (100%)	21 (100%)
Apron	3 (100%)	2 (50%)	11 (52.4%)
Autoclave (or other appropriate equipment for sterilization)	3 (100%)	2 (66.7%)<br>ND* (1)	15 (71.4%)
Incinerator	2 (100%)<br>ND* (1)	1 (33.3%)<br>ND* (1)	11 (52.4%)
Sharps are separated from other waste and disposed of properly.	3 (100%)	3 (75%)	16 (80%)<br>ND* (1)
**Facilities with all minimum infection prevention supplies available**	**1 (50%)<br>ND* (1)**	**0<br>ND* (1)**	**5 (23.8%)**

*****No data

Table A2. DRC: facilities with minimum infection prevention supplies available (n=26)

	Hospital (n=1)	Health center (n=25)
Washing station with soap	1	18 (75%)
Plastic sheeting	1	8 (33.3%)
Non-sterile gloves	1	23 (95.8%)
Sterile gloves	1	19 (79.2%)
Antiseptics	1	23 (95.8%)
Apron	1	17 (70.8%)
Autoclave (or other appropriate equipment for sterilization)	1	12 (50%)
Incinerator	1	16 (64%)
Sharps are separated from other waste and disposed of properly.	1	15 (60%)
**Facilities with all minimum infection prevention supplies available**	**1**	**4 (16%)**

Table A3. South Sudan: facilities with minimum infection prevention supplies available (n=9)

	**Hospital (n=1)**	**Health center (n=8)**
Washing station with soap	1	5
Plastic sheeting	0	4
Non-sterile gloves	1	5
Sterile gloves	1	5
Antiseptics	1	5
Apron	1	4
Autoclave (or other appropriate equipment for sterilization)	1	5
Incinerator	1	6
Sharps are separated from other waste and disposed of properly.	1	7
**Facilities with all minimum infection prevention supplies available**	**0**	**4**
