# Supplementary material for: Progress and gaps in reproductive health services in three humanitarian settings: mixed-methods case studies
Source: Confl Health. 2015 Feb 2;9(Suppl 1):S3. doi: 10.1186/1752-1505-9-S1-S3 (PMC4331815; doi:10.1186/1752-1505-9-S1-S3)
Supplement: Additional file 2 — Appendix B [file 1752-1505-9-S1-S3-S2.pdf]

Appendix B: Functioning family planning (FP) service delivery points, by country and by FP method

Table B1. Burkina Faso: facilities with essential components to provide short- and long-acting FP methods (n=28)

	**Hospital (n=3)**	**Camp health center (n=4)**	**Non-camp health centers (n=21)**
**ORAL CONTRACEPTIVE PILLS**			
OCPs provided in last 3 months (self-reported)	3 (100%)	4 (100%)	20 (95.2%)
Staff trained to provide short-acting FP methods	3 (100%)	3 (100%) ND* (1)	20 (95.2%)
FP counseling available	3 (100%)	4 (100%)	20 (95.2%)
BP cuff	3 (100%)	4 (100%)	19 (90.5%)
Stethoscope	3 (100%)	4 (100%)	21 (100%)
Daily oral contraceptive pills	3 (100%)	4 (100%)	20 (95.2%)
**Functioning OCP service delivery point**	**3 (100%)**	**3 (100%) ND* (1)**	**17 (81%)**
**INJECTABLES**			
Injectables provided in last 3 months (self-reported)	3 (100%)	4 (100%)	20 (95.2%)
Staff trained to provide short-acting FP methods	3 (100%)	3 (100%) ND* (1)	20 (95.2%)
FP counseling available	3 (100%)	4 (100%)	20 (95.2%)
BP cuff	3 (100%)	4 (100%)	19 (90.5%)
Stethoscope	3 (100%)	4 (100%)	21 (100%)
Needles and syringes	3 (100%)	4 (100%)	21 (100%)
Injectable contraceptive (progestin-only)	3 (100%)	4 (100%)	20 (95.2%)
**Functioning injectable service delivery point**	**3 (100%)**	**3 (100%) ND* (1)**	**17 (81%)**
**IUD**			
IUD inserted in last 3 months (self-reported)	3 (100%)	1 (25%)	4 (19%)
Staff trained to provide long-acting methods	3 (100%)	3 (100%) ND* (1)	17 (85%) ND* (1)
FP counseling available	3 (100%)	4 (100%)	20 (95.2%)
Sterile gloves	3 (100%)	4 (100%)	21 (100%)
Speculum	3 (100%)	4 (100%)	20 (95.2%)
Uterine sound	3 (100%)	3 (75%)	5 (23.8%)
Uterine tenaculum	3 (100%)	1 (25%)	6 (30%) ND* (1)
Sponge forceps	3 (100%)	2 (50%)	13 (61.9%)
Antiseptics	3 (100%)	4 (100%)	21 (100%)
IUD (copper or levonorgestrel)	3 (100%)	1 (25%)	10 (47.6%)
**Functioning IUD service delivery point**	**3 (100%)**	**1 (25%)**	**1 (4.8%)**
**IMPLANT**			
Implant inserted in last 3 months (self-reported)	3 (100%)	4 (100%)	19 (90.5%)
Staff trained to provide long-acting methods	3 (100%)	3 (100%) ND* (1)	17 (85%) ND* (1)
FP counseling available	3 (100%)	4 (100%)	20 (95.2%)
Sponge forceps	3 (100%)	2 (50%)	13 (61.9%)
Scalpel handle (No. 3) and blade	3 (100%)	3 (75%)	16 (76.2%)
Needles and syringes	3 (100%)	4 (100%)	21 (100%)
Antiseptics	3 (100%)	4 (100%)	21 (100%)
Implant	3 (100%)	4 (100%)	20 (95.2%)
**Functioning implant service delivery point**	**3 (100%)**	**1 (25%)**	**8 (40%) ND* (1)**

*No data

Table B2. DRC: facilities with essential components to provide short- and long-acting FP methods (n=26)

	Hospital (n=1)	Health center (n=25)
**ORAL CONTRACEPTIVE PILLS**		
OCPs provided in last 3 months (self-reported)	1	18 (72%)
Staff trained to provide short-acting FP methods	1	16 (66.7%) ND* (1)
FP counseling available	1	19 (76%)
BP cuff	1	20 (83.3%) ND* (1)
Stethoscope	1	22 (91.7%) ND* (1)
Daily oral contraceptive pills	1	15 (62.5%) ND* (1)
**Functioning OCP service delivery point**	**1**	**12 (48%)**
**INJECTABLES**		
Injectables provided in last 3 months (self-reported)	1	18 (72%) ND* (1)
Staff trained to provide short-acting FP methods	1	16 (66.7%) ND* (1)
FP counseling available	1	19 (76%)
BP cuff	1	20 (83.3%) ND* (1)
Stethoscope	1	22 (91.7%) ND* (1)
Needles and syringes	1	23 (95.8%) ND* (1)
Injectable contraceptive (progestin-only)	1	13 (54.2%) ND* (1)
**Functioning injectable service delivery point**	**1**	**10 (40%)**
**IUD**		
IUD inserted in last 3 months (self-reported)	1	12 (48%)
Staff trained to provide long-acting methods	1	15 (62.5%) ND* (1)
FP counseling available	1	19 (76%)
Sterile gloves	1	19 (79.2%) ND* (1)
Speculum	1	14 (58.3%) ND* (1)
Uterine sound	1	10 (41.7%) ND* (1)
Uterine tenaculum	1	11 (45.8%) ND* (1)
Sponge forceps	1	11 (45.8%) ND* (1)
Antiseptics	1	23 (95.8%) ND* (1)
IUD (copper or levonorgestrel)	1	11 (45.8%) ND* (1)
**Functioning IUD service delivery point**	**1**	**9 (36%)**
**IMPLANT**		
Implant inserted in last 3 months (self-reported)	1	15 (60%)
Staff trained to provide long-acting methods	1	15 (62.5%) ND* (1)
FP counseling available	1	19 (76%)
Sponge forceps	1	11 (45.8%) ND* (1)
Scalpel handle (No. 3) and blade	1	11 (45.8%) ND* (1)
Needles and syringes	1	23 (95.8%) ND* (1)
Antiseptics	1	23 (95.8%) ND* (1)
Implant	1	13 (54.2%) ND* (1)
**Functioning implant service delivery point**	**1**	**5 (20%)**

*****No data

Table B3. South Sudan: facilities with essential components to provide short- and long-acting FP methods (n=9)

	Hospital (n=1)	Health center (n=8)
**ORAL CONTRACEPTIVE PILLS**		
OCPs provided in last 3 months (self-reported)	0	5
Staff trained to provide short-acting FP methods	1	4 ND* (3)
FP counseling available	1	6
BP cuff	1	8
Stethoscope	1	8
Daily oral contraceptive pills	ND*	3 ND* (1)
**Functioning OCP service delivery point**	**0**	**1**
**INJECTABLES**		
Injectables provided in last 3 months (self-reported)	1	4
Staff trained to provide short-acting FP methods	1	4 ND* (3)
FP counseling available	1	6
BP cuff	1	8
Stethoscope	1	8
Needles and syringes	1	7
Injectable contraceptive (progestin-only)	ND*	3 ND* (1)
**Functioning injectable service delivery point**	**ND**	**1**
**IUD**		
IUD inserted in last 3 months (self-reported)	0	0
Staff trained to provide long-acting methods	0	3
FP counseling available	1	6
Sterile gloves	1	8
Speculum	1	6
Uterine sound	1	4 ND* (1)
Uterine tenaculum	1	4 ND* (1)
Sponge forceps	1	4 ND* (1)
Antiseptics	1	8
IUD (copper or levonorgestrel)	ND*	0 ND* (1)
**Functioning IUD service delivery point**	**0**	**0**
**IMPLANT**		
Implant inserted in last 3 months (self-reported)	0	1
Staff trained to provide long-acting methods	0	3
FP counseling available	1	6
Sponge forceps	1	4 ND* (1)
Scalpel handle (No. 3) and blade	0	5 ND* (2)
Needles and syringes	1	7
Antiseptics	1	8
Implant	ND*	2 ND* (1)
**Functioning implant service delivery point**	**0**	**0**

***No data**
