# Supplementary material for: Progress and gaps in reproductive health services in three humanitarian settings: mixed-methods case studies
Source: Confl Health. 2015 Feb 2;9(Suppl 1):S3. doi: 10.1186/1752-1505-9-S1-S3 (PMC4331815; doi:10.1186/1752-1505-9-S1-S3)
Supplement: Additional file 3 — Appendix C [file 1752-1505-9-S1-S3-S3.pdf]

# Appendix C: Functioning basic and comprehensive emergency obstetric and newborn care (BEmONC & CEmONC) service delivery points, by country

## Table C1. Burkina Faso: facilities with essential components to provide BEmONC & CEmONC[1] (n=28)

| | Hospital (n=3) | Camp health center (n=4) | Non-camp health centers (n=21) |
|---|---|---|---|
| At least one staff trained to provide BEmONC | 3 (100%) | 3 (100%)<br>ND* (1) | 18 (85.7%) |
| Parenteral antibiotics | 3 (100%) | 1 (25%) | 21 (100%) |
| Parenteral uterotonics | 3 (100%) | 1 (25%) | 11 (52.4%) |
| Parenteral anticonvulsants | 3 (100%) | 1 (25%) | 7 (33.3%) |
| Manual removal of placenta | 3 (100%) | 2 (50%) | 10 (47.6%) |
| Removal of retained products | 3 (100%) | 1 (25%) | 1 (4.8%) |
| Assisted vaginal delivery | 2 (66.7%) | 0 | 0 |
| Neonatal resuscitation with bag and mask | 3 (100%) | 1 (25%) | 10 (47.6%) |
| Partograph | 3 (100%) | 3 (75%) | 21 (100%) |
| Blood pressure cuff | 3 (100%) | 4 (100%) | 19 (90.5%) |
| Stethoscope | 3 (100%) | 4 (100%) | 21 (100%) |
| **Functioning BEmONC service delivery point** | **1 (33.3%)** | **0** | **0** |
| At least one staff trained to conduct blood transfusion | 3 (100%) | N/A | N/A |
| Blood transfusion | 3 (100%) | N/A | N/A |
| At least one staff trained to perform caesarean section | 2 (1 ND) | N/A | N/A |
| Caesarean section | 1 (33.3%) | N/A | N/A |
| **Functioning CEmONC service delivery point** | **1 (33.3%)** | N/A | N/A |

*****No data

[1] A facility was classified as providing the signal function if the following criteria were met: self-reported provision of the signal function in the previous three months, at least one provider trained in basic or comprehensive EmONC, presence of minimum essential supplies and equipment for the signal function on the day of the assessment. Hospitals that met the criteria for a comprehensive EmONC facility are not included in the basic EmONC data.

## Table C2. DRC: facilities with essential components to provide BEmONC & CEmONC[1] (n=26)

| | Hospital (n=1) | Health center (n=25) |
|---|---|---|
| At least one staff trained to provide BEmONC | 1 | 16 (69.6%) |
| Parenteral antibiotics | 1 | 2 (8.3%) |
| Parenteral uterotonics | 1 | 4 (16.7%) |
| Parenteral anticonvulsants | 1 | 1 (4%) |
| Manual removal of placenta | 1 | 7 (28%) |
| Removal of retained products | 1 | 11 (44%) |
| Assisted vaginal delivery | 1 | 1 (4%) |
| Neonatal resuscitation with bag and mask | 1 | 5 (20%) |
| Partograph | 1 | 21 (87.5%) ND* (1) |
| Blood pressure cuff | 1 | 20 (83.3%) ND* (1) |
| Stethoscope | 1 | 22 (91.7%) ND* (1) |
| **Functioning BEmONC service delivery point** | **1** | **0** |
| At least one staff trained to conduct blood transfusion | 1 | NA |
| Blood transfusion | 0 | NA |
| At least one staff trained to perform caesarean section | 1 | NA |
| Caesarean section | 1 | NA |
| **Functioning CEmONC service delivery point** | **0** | NA |

*****No data

[1] A facility was classified as providing the signal function if the following criteria were met: self-reported provision of the signal function in the previous three months, at least one provider trained in basic or comprehensive EmONC, presence of minimum essential supplies and equipment for the signal function on the day of the assessment. Hospitals that met the criteria for a comprehensive EmONC facility are not included in the basic EmONC data.

## Table C3. South Sudan: facilities with essential components to provide BEmONC & CEmONC[1] (n=9)

| | **Hospital (n=1)** | **Health center (n=8)** |
|---|---|---|
| At least one staff trained to provide BEmONC | 1 | 3 ND* (3) |
| Parenteral antibiotics | ND* | 3 |
| Parenteral uterotonics | 1 | 3 |
| Parenteral anticonvulsants | 1 | 2 |
| Manual removal of placenta | 1 | 5 |
| Removal of retained products | 1 | 2 ND* (1) |
| Assisted vaginal delivery | 0 | 3 |
| Neonatal resuscitation with bag and mask | 1 | 5 |
| Partograph | 0 | 4 |
| Blood pressure cuff | 1 | 8 |
| Stethoscope | 1 | 8 |
| **Functioning BEmONC service delivery point** | **0** | **1 ND* (1)** |
| At least one staff trained to conduct blood transfusion | 1 | 3 ND* (3) |
| Blood transfusion | 1 | NA |
| At least one staff trained to perform caesarean section | 1 | NA |
| Caesarean section | 1 | NA |
| **Functioning CEmONC service delivery point** | **0** | NA |

*****No data

[1] A facility was classified as providing the signal function if the following criteria were met: self-reported provision of the signal function in the previous three months, at least one provider trained in basic or comprehensive EmONC, presence of minimum essential supplies and equipment for the signal function on the day of the assessment. Hospitals that met the criteria for a comprehensive EmONC facility are not included in the basic EmONC data.
