# Supplementary material for: Progress and gaps in reproductive health services in three humanitarian settings: mixed-methods case studies
Source: Confl Health. 2015 Feb 2;9(Suppl 1):S3. doi: 10.1186/1752-1505-9-S1-S3 (PMC4331815; doi:10.1186/1752-1505-9-S1-S3)
Supplement: Additional file 4 — Appendix D [file 1752-1505-9-S1-S3-S4.pdf]

Appendix D: Facilities able to provide essential elements of newborn care, by country

Table D1. Burkina Faso: facilities with components to provide essential elements of newborn care (n=28)

	Hospital (n=3)	Camp health center (n=4)	Non-camp health center (n=21)
Neonatal resuscitation with appropriate bag and mask performed in last 3 months	3 (100%)	3 (75%)	11 (52.4%)
At least one provider trained to provide:			
Newborn infection management (including injectable antibiotics)	3 (100%)	3 (75%)	12 (57.1%)
Thermal care (including immediate drying and skin-to-skin care)	3 (100%)	3 (75%)	17 (81%)
Sterile cord cutting and appropriate cord care	3 (100%)	4 (100%)	17 (81%)
Kangaroo care for low birth weight babies	3 (100%)	4 (100%)	18 (85.7%)
Special delivery care practices to prevent mother-to-child transmission of HIV	3 (100%)	4 (100%)	20 (95.2%)
Breastfeeding (early and exclusive)	2 (66.7%)	3 (75%)	15 (71.4%)
Partograph	3 (100%)	3 (75%)	21 (100%)
Resuscitation bag and infant face mask	3 (100%)	1 (25%)	12 (57.1%)
Infant scale	3 (100%)	3 (75%)	21 (100%)
Fetoscope	3 (100%)	4 (100%)	21 (100%)
Corticosteroids (dexamethasone)	3 (100%)	3 (75%)	4 (20%)<br>ND* (1)
Ampicillin injectable	3 (100%)	4 (100%)	21 (100%)
Gentamycin injectable	3 (100%)	3 (75%)	21 (100%)
Ceftriaxone injectable	3 (100%)	4 (100%)	21 (100%)
**Facilities with all minimum elements of essential newborn care**	**2 (66.7%)**	**1 (25%)**	**2 (9.5%)**

*****No data

Table D2. DRC: facilities with components to provide essential elements of newborn care (n=26)

	Hospital (n=1)	Health center (n=25)
Neonatal resuscitation with appropriate bag and mask performed in last 3 months	1	6 (25%) ND*(1)
At least one provider trained to provide:		
Newborn infection management (including injectable antibiotics)	1	8 (34.8%) ND* (2)
Thermal care (including immediate drying and skin-to-skin care)	1	13 (56.5%) ND* (2)
Sterile cord cutting and appropriate cord care	1	20 (87%) ND* (2)
Kangaroo care for low birth weight babies	1	18 (78.3%) ND* (2)
Special delivery care practices to prevent mother-to-child transmission of HIV	1	10 (43.5%) ND* (2)
Breastfeeding (early and exclusive)	1	14 (60.7%) ND*(2)
Partograph	1	21 (87.5%) ND* (1)
Resuscitation bag and infant face mask	1	8 (33.3%) ND* (1)
Infant scale	1	14 (58.3%) ND* (1)
Fetoscope	1	17 (70.8%) ND* (1)
Corticosteroids (dexamethasone)	1	5 (20.8%) ND* (1)
Ampicillin injectable	1	7 (29.2%) ND* (1)
Gentamycin injectable	1	6 (25%) ND* (1)
Ceftriaxone injectable	1	3 (13%) ND* (2)
**Facilities with all minimum elements of essential newborn care**	**1**	**0**

***No data

Table D3. South Sudan: facilities with components to provide essential elements of newborn care (n=9)

	Hospital (n=1)	Health center (n=8)
Neonatal resuscitation with appropriate bag and mask performed in last 3 months	1	5
At least one provider trained to provide:		
Newborn infection management (including injectable antibiotics)	0	5
Thermal care (including immediate drying and skin-to-skin care)	0	6
Sterile cord cutting and appropriate cord care	1	6
Kangaroo care for low birth weight babies	0	6
Special delivery care practices to prevent mother-to-child transmission of HIV	1	3
Breastfeeding (early and exclusive)	0	7
Partograph	0	4
Resuscitation bag and infant face mask	1	5
Infant scale	1	5
Fetoscope	1	7
Corticosteroids (dexamethasone)	ND*	4 ND* (1)
Ampicillin injectable	ND*	4 ND* (1)
Gentamycin injectable	ND*	6 ND* (1)
Ceftriaxone injectable	ND*	6 ND* (1)
**Facilities with all minimum elements of essential newborn care**	**0**	**2**

***No data
