# Supplementary material for: Progress and gaps in reproductive health services in three humanitarian settings: mixed-methods case studies
Source: Confl Health. 2015 Feb 2;9(Suppl 1):S3. doi: 10.1186/1752-1505-9-S1-S3 (PMC4331815; doi:10.1186/1752-1505-9-S1-S3)
Supplement: Additional file 5 — Appendix E [file 1752-1505-9-S1-S3-S5.pdf]

Appendix E: Functioning post-abortion care (PAC) service delivery points, by country

Table E1. Burkina Faso: facilities with essential components to provide PAC (n=28)

	Hospital (n=3)	Camp health center (n=4)	Non-camp health center (n=21)
**COUNSELING & FAMILY PLANNING**			
PAC counseling is available	3 (100%)	4 (100%)	21 (100%)
Family planning is offered to all clients who receive PAC services before they are discharged from the facility	3 (100%)	4 (100%)	21 (100%)
**Facilities with minimum essential elements to provide counseling and family planning for clients who receive PAC services**	3 (100%)	4 (100%)	21 (100%)
**PAC WITH MANUAL VACUUM ASPIRATION (MVA)**			
PAC performed in last 3 months using MVA	3 (100%)	1 (25%)	4 (19%)
At least 1 trained staff to provide PAC	3 (100%)	2 (100%)<br>ND* (2)	16 (94.12%)<br>ND* (4)
Vaginal speculum	3 (100%)	4 (100%)	20 (95.2%)
Sponge forceps	3 (100%)	2 (50%)	13 (61.9%)
Uterine tenaculum	3 (100%)	1 (25%)	6 (30%)<br>ND* (1)
MVA syringe, adapters and cannulae	3 (100%)	1 (33.3%)<br>ND* (1)	4 (19%)
Antiseptic solution	3 (100%)	4 (100%)	21 (100%)
Non-sterile Gloves	3 (100%)	4 (100%)	21 (100%)
Oxytocin	3 (100%)	4 (100%)	21 (100%)
Needles and syringes	3 (100%)	4 (100%)	21 (100%)
**Facilities with minimum essential elements to provide PAC with MVA and provided in the previous three months**	**3 (100%)**	**1 (25%)**	**0 (0%)**
**PAC WITH MISOPROSTOL**			
PAC performed in last 3 months using misoprostol	2 (66.7%)	1 (25%)	0
At least 1 trained staff to provide PAC	3 (100%)	2 (100%)<br>ND* (2)	16 (94.1%)<br>ND* (4)
Misoprostol	0	0	1 (4.8%)
**Facilities with minimum essential elements to provide PAC with misoprostol and provided in the previous three months**	**0**	**0**	**0**
**PROVISION OF PAC TO AN ACCEPTABLE STANDARD**			
FP is offered to all PAC clients	3 (100%)	4 (100%)	21 (100%)
PAC with MVA	3 (100%)	1 (25%)	0 (0%)
PAC with misoprostol *(optional)*	0	0	0
**Functioning PAC service delivery point**	**3 (100%)**	**1 (25%)**	**0 (0%)**

*****No data

Table E2. DRC: facilities with essential components to provide PAC (n=26)

	Hospital (n=1)	Health center (n=26)
**COUNSELING & FAMILY PLANNING**		
PAC counseling is available	1	17 (68%)
Family planning is offered to all clients who receive PAC services before they are discharged from the facility	1	13 (54.2%) ND* (1)
**Facilities with minimum essential elements to provide counseling and family planning for clients who receive PAC services**	**1**	**13 (52%)**
**PAC WITH MANUAL VACUUM ASPIRATION (MVA)**		
PAC performed in last 3 months using MVA	1	11 (45.8%) ND* (1)
At least 1 trained staff to provide PAC	1	14 (58.3%) ND* (1)
Vaginal speculum	1	14 (58.3%) ND* (1)
Sponge forceps	1	11 (45.8%) ND* (1)
Uterine tenaculum	1	11 (45.8%) ND* (1)
MVA syringe, adapters and cannulae	1	11 (45.8%) ND* (1)
Antiseptic solution	1	23 (95.8%) ND* (1)
Non-sterile Gloves	1	23 (95.8%) ND* (1)
Oxytocin	1	20 (83.3%) ND* (1)
Needles and syringes	1	23 (95.8%) ND* (1)
**Facilities with minimum essential elements to provide PAC with MVA and provided in the previous three months**	**1**	**9 (38%)**
**PAC WITH MISOPROSTOL**		
PAC performed in last 3 months using misoprostol	1	2 (8%)
At least 1 trained staff to provide PAC	1	14 (58.3%) ND* (1)
Misoprostol	1	2 (8.3%) ND* (1)
**Facilities with minimum essential elements to provide PAC with misoprostol and provided in the previous three months**	**1**	**2 (8%)**
**PROVISION OF PAC TO AN ACCEPTABLE STANDARD**		
FP is offered to all PAC clients	1	13 (52%) ND* (1)
PAC with MVA	1	9 (37.5%) ND* (1)
PAC with misoprostol *(optional)*	1	2 (8%) ND* (1)
**Functioning PAC service delivery point**	**1**	**11 (44%)**

*****No data

Table E3. South Sudan: facilities with essential components to provide PAC (n=9)

	**Hospital (n=1)**	**Health center (n=8)**
**COUNSELING & FAMILY PLANNING**		
PAC counseling is available	1	4
Family planning is offered to all clients who receive PAC services before they are discharged from the facility	1	3
**Facilities with minimum essential elements to provide counseling and family planning for clients who receive PAC services**	**1**	**3**
**PAC WITH MANUAL VACUUM ASPIRATION (MVA)**		
PAC performed in last 3 months using MVA	1	3
At least 1 trained staff to provide PAC	1	3 ND* (3)
Vaginal speculum	1	6
Sponge forceps	1	4 ND* (1)
Uterine tenaculum	1	4 ND* (1)
MVA syringe, adapters and cannulae	1	4
Antiseptic solution	1	8
Non-sterile Gloves	1	8
Oxytocin	1	5
Needles and syringes	1	7
**Facilities with minimum essential elements to provide PAC with MVA and provided in the previous three months**	**1**	**1**
**PAC WITH MISOPROSTOL**		
PAC performed in last 3 months using misoprostol	1	3
At least 1 trained staff to provide PAC	1	3 ND* (3)
Misoprostol	ND*	3 ND* (1)
**Facilities with minimum elements to provide PAC with misoprostol and provided in the previous three months**	**ND***	**1**
**PROVISION OF PAC TO AN ACCEPTABLE STANDARD**		
FP is offered to all PAC clients	1	3
PAC with MVA	1	1
PAC with misoprostol *(optional)*	ND*	1
**Functioning PAC service delivery point**	**1**	**1**

*****No data
