# Supplementary material for: Progress and gaps in reproductive health services in three humanitarian settings: mixed-methods case studies
Source: Confl Health. 2015 Feb 2;9(Suppl 1):S3. doi: 10.1186/1752-1505-9-S1-S3 (PMC4331815; doi:10.1186/1752-1505-9-S1-S3)
Supplement: Additional file 6 — Appendix F [file 1752-1505-9-S1-S3-S6.pdf]

Appendix F: Facilities able to provide selected elements of clinical management of rape (CMoR), by country

Table F1. Burkina Faso: Facilities able to provide selected elements of clinical management of rape (n=28)

	**Hospital (n=3)**	**Camp health center (n=4)**	**Non-camp health center (n=21)**
**POST-EXPOSURE PROPHYLAXIS (PEP)**			
Provision of PEP for CMoR in last 3 months	2 (66.7%)	3 (75%)	5 (23.8%)
PEP	3 (100%)	0	0
**Facilities with minimum elements to provide PEP**	**2 (66.7%)**	**0**	**0**
**EMERGENCY CONTRACEPTION**			
Provision of EC for CMoR in last 3 months	2 (66.7%)	4 (100%)	7 (33.3%)
EC	0	2 (66.7%) ND* (1)	7 (33.3%)
**Facilities with minimum elements to provide EC**	**0**	**2 (66.7%) ND* (1)**	**6 (28.6%)**
**ANTIBIOTICS FOR SEXUALLY TRANSMITTED INFECTIONS (STIs)**			
Provision of antibiotics for presumptive treatment of STIs for CMoR	2 (66.7%)	4 (100%)	9 (42.9%)
Gentamycin	3 (100%)	3 (75%)	21 100%)
Ceftriaxone	3 (100%)	4 (100%)	21 100%)
Injectable metronidazole	3 (100%)	2 (50%)	21(100%)
**Facilities with minimum elements to antibiotics for STIs**	**2 (66.7%)**	**2 (50%)**	**9 (42.9%)**

*No data

Table F2. DRC: Facilities able to provide selected elements of clinical management of rape (n=26)

	**Hospital (n=1)**	**Health center (n=25)**
**POST-EXPOSURE PROPHYLAXIS (PEP)**		
Provision of PEP for CMoR in last 3 months	1	17 (68%)
PEP	1	11 (45.8%) ND* (1)
**Facilities with minimum elements to provide PEP**	**1**	**10 (40%)**
**EMERGENCY CONTRACEPTION**		
Provision of EC for CMoR in last 3 months	1	17 (68%)
EC	1	13 (54.2%) ND* (1)
**Facilities with minimum elements to provide EC**	**1**	**12 (48%)**
**ANTIBIOTICS FOR SEXUALLY TRANSMITTED INFECTIONS (STIs)**		
Provision of antibiotics for presumptive treatment of STIs for CMoR	1	20 (80%)
Gentamycin	1	10 (40%) ND* (1)
Ceftriaxone	1	12 (48%)
Injectable metronidazole	1	2 (8%)
**Facilities with minimum elements to provide antibiotics for STIs**	**1**	**2 (8%)**

*****No data

Table F3. South Sudan: Facilities able to provide selected elements of clinical management of rape (n=9)

	**Hospital (n=1)**	**Health center (n=8)**
**POST-EXPOSURE PROPHYLAXIS (PEP)**		
Provision of PEP for CMoR in last 3 months	0	2
PEP	0	1 ND* (1)
**Facilities with minimum elements to provide PEP**	0	1 ND* (1)
**EMERGENCY CONTRACEPTION**		
Provision of EC for CMoR in last 3 months	0	2
EC	ND* (1)	2 ND* (2)
**Facilities with minimum elements to provide EC**	**0**	**1 ND* (1)**
**ANTIBIOTICS FOR SEXUALLY TRANSMITTED INFECTIONS (STIs)**		
Provision of antibiotics for presumptive treatment of STIs for CMoR	0	4
Gentamycin	0	6 ND* (1)
Ceftriaxone	0	6 ND* (1)
Injectable metronidazole	0	4 ND* (1)
**Facilities with minimum elements to provide antibiotics for STIs**	**0**	**2**

*****No data
