# Supplementary material for: Progress and gaps in reproductive health services in three humanitarian settings: mixed-methods case studies
Source: Confl Health. 2015 Feb 2;9(Suppl 1):S3. doi: 10.1186/1752-1505-9-S1-S3 (PMC4331815; doi:10.1186/1752-1505-9-S1-S3)
Supplement: Additional file 7 — Appendix G [file 1752-1505-9-S1-S3-S7.pdf]

Appendix G: Facilities with essential supplies for diagnosis and treatment of sexually transmitted infections (STIs) and prevention of mother-to-child transmission of HIV (PMTCT), by country

Table G1. Burkina Faso: Facilities with essential supplies for diagnosis and treatment of STIs and for PMTCT (n=28)

	**Hospital (n=3)**	**Camp health center (n=4)**	**Non-camp health center (n=21)**
**DIAGNOSIS & TREATMENT OF STIs**			
Performed syndromic or laboratory diagnosis and treatment of STIs in last 3 months	2 (67%)	4 (100%)	21 (100%)
Gentamycin	3 (100%)	3 (75%)	21 (100%)
Ceftriaxone	3 (100%)	4 (100%)	21 (100%)
Injectable metronidazole	3 (100%)	2 (50%)	21 (100%)
**Facilities with essential supplies for diagnosis and treatment of STIs**	**2 (67%)**	**1 (25%)**	**21 (100%)**
**PROVISION OF PMTCT**			
Administered ARVs to HIV+ mothers in maternity in the last 3 months	3 (100%)	1 (33.3%)<br>ND* (1)	21 (100%)
Administered ARVs to newborns born to HIV+ mothers in maternity in the last 3 months	3 (100%)	1 (25%)	21 (100%)
ARVs for the mother	3 (100%)	3 (75%)	19 (90.5%)
ARVs for the infant	3 (100%)	3 (75%)	18 (90%)<br>ND* (1)
**Facilities with essential supplies to provide PMTCT**	**3 (100%)**	**1 (25%)**	**18 (90%)<br>ND* (1)**

*****No data

Table G2. DRC: Facilities with essential supplies for diagnosis and treatment of STIs and for PMTCT (n=26)

	Hospital (n=1)	Health center (n=25)
**DIAGNOSIS & TREATMENT OF STIs**		
Performed syndromic or laboratory diagnosis and treatment of STIs in last 3 months	1 (100%)	22 (88%)
Gentamycin	1 (100%)	6 (25%) ND* (1)
Ceftriaxone	1 (100%)	3 (13%) ND* (2)
Injectable metronidazole	1 (100%)	3 (13%) ND* (2)
**Facilities with essential supplies for diagnosis and treatment of STIs**	**1 (100%)**	**2 (9%) ND* (2)**
**PROVISION OF PMTCT**		
Administered ARVs to HIV+ mothers in maternity in the last 3 months	1	0
Administered ARVs to newborns born to HIV+ mothers in maternity in the last 3 months	1	1 (4%)
ARVs for the mother	1	1 (4%) ND* (1)
ARVs for the infant	1	0 ND* (1)
**Facilities with essential supplies to provide PMTCT**	**1**	**0**

***No data**

Table G3. South Sudan: Facilities with essential supplies for diagnosis and treatment of STIs and for PMTCT (n=9)

	Hospital (n=1)	Health center (n=8)
**DIAGNOSIS & TREATMENT OF STIs**		
Performed syndromic or laboratory diagnosis and treatment of STIs in last 3 months	1	7
Gentamycin	0	6 ND* (1)
Ceftriaxone	0	6 ND* (1)
Injectable metronidazole	0	4 ND* (1)
**Facilities with essential supplies for diagnosis and treatment of STIs**	**0**	**3**
**PROVISION OF PMTCT**		
Administered ARVs to HIV+ mothers in maternity in the last 3 months	0	0
Administered ARVs to newborns born to HIV+ mothers in maternity in the last 3 months	0	0
ARVs for the mother	ND*	0 ND* (1)
ARVs for the infant	ND*	0 ND* (1)
**Facilities with essential supplies to provide PMTCT**	**0**	**0**

***No data**
