# Supplementary material for: Progress and gaps in reproductive health services in three humanitarian settings: mixed-methods case studies
Source: Confl Health. 2015 Feb 2;9(Suppl 1):S3. doi: 10.1186/1752-1505-9-S1-S3 (PMC4331815; doi:10.1186/1752-1505-9-S1-S3)
Supplement: Additional file 8 — Appendix H [file 1752-1505-9-S1-S3-S8.pdf]

# Appendix H: Knowledge and attitudes of health care providers toward reproductive health services, selected questions

## Table H1. DRC and South Sudan: mean reproductive health knowledge scores among health care providers*[1] (n=31)

| | DRC (n=13) | South Sudan (n=18) |
|---|---|---|
| 1. For a woman in labor, what observations do you make as you monitor her progress?<br>a. Fetal heartbeat<br>b. Color of amniotic fluid<br>c. Degree of molding<br>d. Dilatation of the cervix<br>e. Descent of the head<br>f. Uterine contractions<br>g. Maternal blood pressure<br>h. Maternal temperature<br>i. Maternal pulse | 5.21 / 9 | 6.33 / 9 |
| 2. When a woman arrives at the facility with heavy bleeding or develops severe bleeding after giving birth, what do you look for?<br>a. Signs of shock (dizziness, low blood pressure)<br>b. Amount of external blood<br>c. Signs of anemia<br>d. Damage to the genital tract<br>e. Whether the uterus is contracted<br>f. Retained products or retained placenta<br>g. Full bladder | 2.93 / 7 | 3.44 / 7 |
| 3. When a woman develops heavy bleeding after delivery, what do you do?<br>a. Massage the fundus<br>b. Give ergometrine or oxytocin (intravenous (IV) or intramuscular)<br>c. Begin IV fluids<br>d. Empty full bladder<br>e. Take blood for hemoglobin and cross-matching<br>f. Examine woman for lacerations<br>g. Manually remove retained products<br>h. Refer | 2.71 / 8 | 3.5 / 8 |
| 4. When a woman who just gave birth has not delivered the placenta, what do you do?<br>a. Empty the bladder<br>b. Check for signs of separation of placenta before controlled cord traction<br>c. Give or repeat oxytocin<br>d. Do manual removal of the placenta<br>e. Administer IV fluids<br>f. Monitor vital signs for shock and act<br>g. Check that uterus is well contracted<br>h. Determine blood type and cross-match<br>i. Prepare operating theater<br>j. Refer | 3 / 10 | 2.44 / 10 |

| | | |
|---|---|---|
| 5. The last time you delivered a baby, what immediate care did you give the newborn?<br>a. Clean the baby’s mouth before the shoulder comes out<br>b. Clean the baby’s mouth, face, and nose<br>c. Ensure the baby is breathing<br>d. Ensure the baby is dry<br>e. Observe for color<br>f. Ensure baby is kept warm (skin-to-skin)<br>g. Administer prophylaxis for the eyes<br>h. Weigh the baby<br>i. Care for the umbilical cord<br>j. Initiate breastfeeding within the first 30 minutes<br>k. Evaluate/examine newborn within the first hour | 5 / 11 | 6.78 / 11 |
| 6. What are the signs and symptoms of infection, or sepsis, in the newborn?<br>a. Less movement (poor muscle tone)<br>b. Poor or no breastfeeding<br>c. Hypothermia or hyperthermia<br>d. Restlessness or irritability<br>e. Difficulty breathing or fast breathing<br>f. Deep jaundice<br>g. Severe abdominal distention | 3.14 / 7 | 2.89 / 7 |
| 7. When a newborn weighs less than 2.5kgs, what special care do you provide?<br>a. Make sure the baby is warm (skin-to-skin/kangaroo technique)<br>b. Provide extra support to the mother to establish breastfeeding<br>c. Monitor ability to breastfeed<br>d. Monitor baby for the first 24 hours<br>e. Ensure infection prevention | 1.64 / 5 | 3.17 / 5 |
| 8. Which FP methods can a woman use immediately post-partum?<br>a. IUD<br>b. Tubal ligation<br>c. Condoms<br>d. Lactational Amenorrhea Method | 1.43 / 4 | 1 / 4 |
| 9. Which FP methods can a woman who is breastfeeding begin using 6 weeks after delivery?<br>a. IUD<br>b. Tubal ligation<br>c. Condoms<br>d. Progestin-only pills | 2.07 / 4 | 1.39 / 4 |
| 10. When you counsel a woman for FP, what do you do/discuss?<br>a. Ask whether she has used FP before<br>b. Ask about her reproductive goals<br>c. Check for medical complications<br>d. Tell her about dual protection<br>e. Tell her about all FP methods | 2 / 5 | 2.17 / 5 |

| | | |
|---|---|---|
| 11. What are the immediate complications of an unsafe abortion?<br>a. Sepsis<br>b. Bleeding<br>c. Genital injuries<br>d. Abdominal injuries<br>e. Shock | 2.71 / 5 | 3 / 5 |
| 12. When you see a woman with complications from an unsafe or incomplete abortion, what do you do?<br>a. Do a vaginal exam<br>b. Assess vaginal bleeding<br>c. Assess vital signs<br>d. Begin IV fluids<br>e. Begin antibiotics<br>f. Do (manual/electric) vacuum aspiration<br>g. Do dilatation with curettage or evacuation<br>h. Provide misoprostol<br>i. Provide counseling<br>j. Refer | 3.93 / 10 | 4.06 / 10 |
| 13. What information do you give patients who were treated for an incomplete or unsafe abortion?<br>a. Information on how to prevent STIs/HIV<br>b. Information about when a woman can conceive again<br>c. Counseling on family planning and services<br>d. Refer for FP or provide FP methods<br>e. Information on social support<br>f. Information about the consequences of an unsafe abortion | 2 / 6 | 2.39 / 6 |
| 14. What do you do when someone presents with signs of an STI?<br>a. Diagnose and provide antibiotics according to the syndromic approach<br>b. Counsel on contact tracing<br>c. Explain how to use and provide condoms<br>d. Counsel on HIV and offer VCT<br>e. Refer | 2.43 / 5 | 2.83 / 5 |
| 15. When a woman presents after a rape, what do you do?<br>a. Encourage her to report to police<br>b. Facilitate filling out the police report<br>c. Take history and do an examination<br>d. Take forensic evidence<br>e. Counsel for pre- and post-HIV testing<br>f. Counsel about pregnancy prevention<br>g. Provide emergency contraception<br>h. Provide post-exposure prophylaxis for HIV<br>i. Provide antibiotics to prevent STIs<br>j. Request that she do urine, vaginal smear/swabs, and/or blood exams<br>k. Refer | 4.71 / 11 | 4.28 / 11 |

*Scores are out of the total possible score for each item, which is based on a pre-defined list of essential steps for delivering good quality care.

[1] Due to challenges in data management, means from Burkina Faso could not be calculated. Instead, the descriptive summaries of providers' responses were analyzed.

**Table H2. DRC and South Sudan: mean reproductive health attitude scores among health care providers*[1] (n=31)**

| | DRC (n=13) | South Sudan (n=18) |
|---|---|---|
| 1. Visiting a health facility to check on a pregnancy's progress is a good idea for a pregnant woman. | 3.86 | 3.94 |
| 2. Delivering a baby at a health facility is safer than delivering a baby at home. | 3.93 | 3.89 |
| 3. Within the couple, both the wife and the husband should have equal say in important decisions. | 3.43 | 3.61 |
| 4. The more children a mother has, the more respected she is in the community. | 2.43 | 3.28 |
| 5. Men should be responsible for choosing how many children their wife/wives will have. | 2.79 | 3.28 |
| 6. Family planning should be available to every woman who wants to use a method. | 3.36 | 3.67 |
| 7. A woman should be able to obtain a family planning method without her husband's presence. | 2.57 | 2.72 |
| 8. IUDs can be a good method for women who have no children. | 2.07 | 2.06 |
| 9. Adolescent, unmarried girls should be allowed to obtain family planning if they want. | 3.14 | 3.22 |
| 10. Young unmarried men and women need to know how to prevent pregnancies. | 3.57 | 3.61 |
| 11. Young unmarried women should be required to get their parents' consent in order to receive a family planning method. | 2.86 | 3 |
| 12. Young unmarried men and women should be educated about sex and reproduction | 3.86 | 3.61 |

*All scores are based on Likert scale responses to each statement: strongly agree (4), agree (3), disagree (2), and strongly disagree (1). Statements 4, 5, and 11 were reverse coded. All items are scored out of a total possible score of 4 with higher means signalling attitudes that promote equitable and good quality RH services.

[1] Due to challenges in data management, means from Burkina Faso could not be calculated. Instead, the descriptive summaries of providers' responses were analyzed.
